# The Correlation Between the Types of Initial Bacterial Infection and Clinical Prognosis in Patients With Septic AKI

**DOI:** 10.3389/fmed.2021.800532

**Published:** 2022-01-27

**Authors:** Tian Li, Jing yuan Liu, Jing feng Liu, Meili Duan, Ang Li

**Affiliations:** ^1^Department of Critical Care Medicine, Beijing Ditan Hospital, Capital Medical University, Beijing, China; ^2^Department of Critical Care Medicine, Beijing Friendship Hospital, Capital Medical University, Beijing, China

**Keywords:** acute kidney injury, gram-positive bacteria, gram-negative bacteria, clinical outcome, sepsis

## Abstract

**Background:**

Acute kidney injury (AKI) is a common complication of sepsis and is an independent risk factor for mortality. It is unclear whether different bacteria play different roles in the occurrence and development of sepsis-associated AKI (S-AKI). We observed the clinical characteristics and outcomes of patients that have types of bacterial infection, and different infections sites before the occurrence of AKI, respectively.

**Methods:**

Data of patients who were diagnosed with sepsis and later developed AKI from 2008 to 2019 were retrieved from the MIMIC-IV 1.0 database. Patients were first divided into the two groups according to the bacterial culture results obtained prior to AKI occurrence: bacterial cultured positive (*N* = 1,785) and bacterial cultured negative (*N* = 8,777). Patients with bacteria culture positive were divided into culture bacteria Gram-positive (CGP, *N* = 1248) and Gram-negative (CGN, *N* = 537) groups.

**Results:**

Overall, 1,785 patients were included in the present analysis. The patients in CGN group were older (70 vs. 66, *p* < 0.001), had lower body mass index (BMI) (27.0 vs. 28.4, *p* < 0.001), higher acute physiology III (APS III) score (63.0 vs. 58, *p* = 0.001), shorter time from positive microbial culture to diagnosis of AKI (2.94 vs. 3.16 days, *p* = 0.013) and longer intensive care unit (ICU) stay time (5.94 vs. 4.77 days, *p* < 0.001) compared with those in the CGP group (*n* = 1,248). In the culture gram-negative bacteria in patients with positive blood cultures (CGNb) group, the rate of vasopressors using (73.1 vs. 56.4%, *P* = 0.007), the Sequential Organ Failure Assessment (SOFA) score (10 vs. 9, *p* = 0.005), and the level of lactate (3.7 vs. 2.5, *p* = 0.001) were higher than those in the culture gram-positive bacteria in patients with positive blood cultures (CGPb) group. The time from positive microbial culture to the diagnosis of AKI was shorter (2.23 vs. 3 days, *p* = 0.001) in the CGNb group. However, there was no significant difference in the continuous renal replacement treatment (CRRT) application or short-term mortality rates between CGN and CGP groups.

**Conclusion:**

The Gram types of bacteria cultured prior to S-AKI occurrence was not related to AKI stage, CRRT application, and short-term mortality. Compared with the Gram-positive bacterial infections, Gram-negative bacterial infections take a shorter time to develop into AKI, and had a higher disease severity score.

## Introduction

Sepsis is a life-threatening condition characterized by an organ dysfunction caused by an unregulated host response to infection. Sepsis is a major cause of morbidity and mortality globally; the Global Burden of Disease Study showed that in 2017, sepsis-related deaths represented 19.7% of all global deaths (1).

Acute kidney injury (AKI) is a common complication of sepsis that easily develops into chronic kidney disease (CKD) and is an independent risk factor for mortality (2, 3). Sepsis has a complicated pathophysiological process; consequentially, the pathophysiological mechanism of sepsis-associated AKI (S-AKI) is significantly different from other types of AKI.

Recently, there have been several studies on the mechanism of S-AKI. Some studies suggest that S-AKI is caused by traditional ischemia-reperfusion (4, 5) whereas other reports suggest that pathogen-associated molecular patterns and damage-associated molecular patterns may be responsible (6). Sepsis-causing pathogens include bacteria, fungi, and viruses, although sepsis is mostly caused by bacterial infection. In basic research, there are several widely used models of S-AKI induced by bacteria or isolated bacterial pathogenic substances. Common sepsis models include intravenous infusion of *Escherichia coli* into pig or sheep (7, 8), intraperitoneal injection of bacterial lipopolysaccharide (9, 10), *Staphylococcus aureus* implantation in the bronchus or injection into the abdominal cavity (11, 12), and cecal ligation and perforation (13). Despite the various animal models of sepsis and ongoing basic research, the pathophysiology of S-AKI remains unclear. Additionally, it is unknown whether different bacteria play different roles in the occurrence and development of S-AKI. We sought to examine the relationship between bacterial cultures isolated from patients with sepsis and their clinical outcomes using the patient data extracted from the MIMIC-IV database.

## Methods

### Study Design and Population

This was a retrospective, observational study, and all data were extracted from a database of patients admitted to the Beth Israel Deaconess Medical Center (MIMIC-IV version 1.0) from 2008 to 2019 (14). Individuals who complete the Collaborative Institutional Training Initiative examination can access the database (all data were retrieved by Tian Li, certification number 40063205). Data of patients who were aged >18 years, admitted to the intensive care unit (ICU) for the first time, and of the ICU stay time ≥ 24 h were included in the present analysis.

First, patients with sepsis were selected according to the criteria of sepsis 3.0 (sepsis is defined as the earliest time at which a patient had [Sequential Organ Failure Assessment] SOFA ≥ 2 and suspicion of infection). Of these patients, patients with AKI were selected according to the Kidney Disease Improving Global Outcome (KDIGO) criteria. Next, the inclusion criteria were: (1) the time diagnosed sepsis was earlier than the time diagnosis AKI; and (2) the microbiological item is not empty before the occurrence of AKI. The exclusions were: (1) fungal and viral infection; and (2) loss of laboratory results. The code used for data extraction is available at Github (https://github.com/MIT-LCP/mimic-IV).

From the MIMIC-IV database, we first extracted baseline patient data from the admissions and ICU records. Next, we extracted laboratory test results, and finally, extracted pathogenic bacteria data from microbiological events. Patients were then divided into two groups according to the results of their first bacterial positive culture: culture Gram-positive (CGP) and culture Gram-negative (CGN) group. In the case of multiple infection sites, patients were first classified according to blood culture, then, sputum culture, and finally, urine culture results.

### Statistical Analysis

For categorical variables, frequency and percentage were used for the statistical description, and chi square test or Fisher's exact test were used for inter-group comparison. For continuous variables, the mean and SD are used for the statistical description if they conform to the normal distribution, the *t*-test or analysis of variance are used for inter-group comparison, the median and interquartile spacing are used for the statistical description if they do not conform to the normal distribution, and the nonparametric test is used for inter-group comparison. Kaplan–Meier curve was used to present and compare the risk of ICU death between different groups. Multivariate Cox proportional hazard models based on baseline demographics, and the clinical characteristics of admission were developed to test the risk factors of death in ICU. Statistical analysis was performed using R software 4.0.3 (R Foundation for Statistical Computing, Vienna, Austria). A two-sided *p* < 0.05 was considered statistically significant.

## Results

### Clinical Characteristics of the Patients

**Figure 1:**
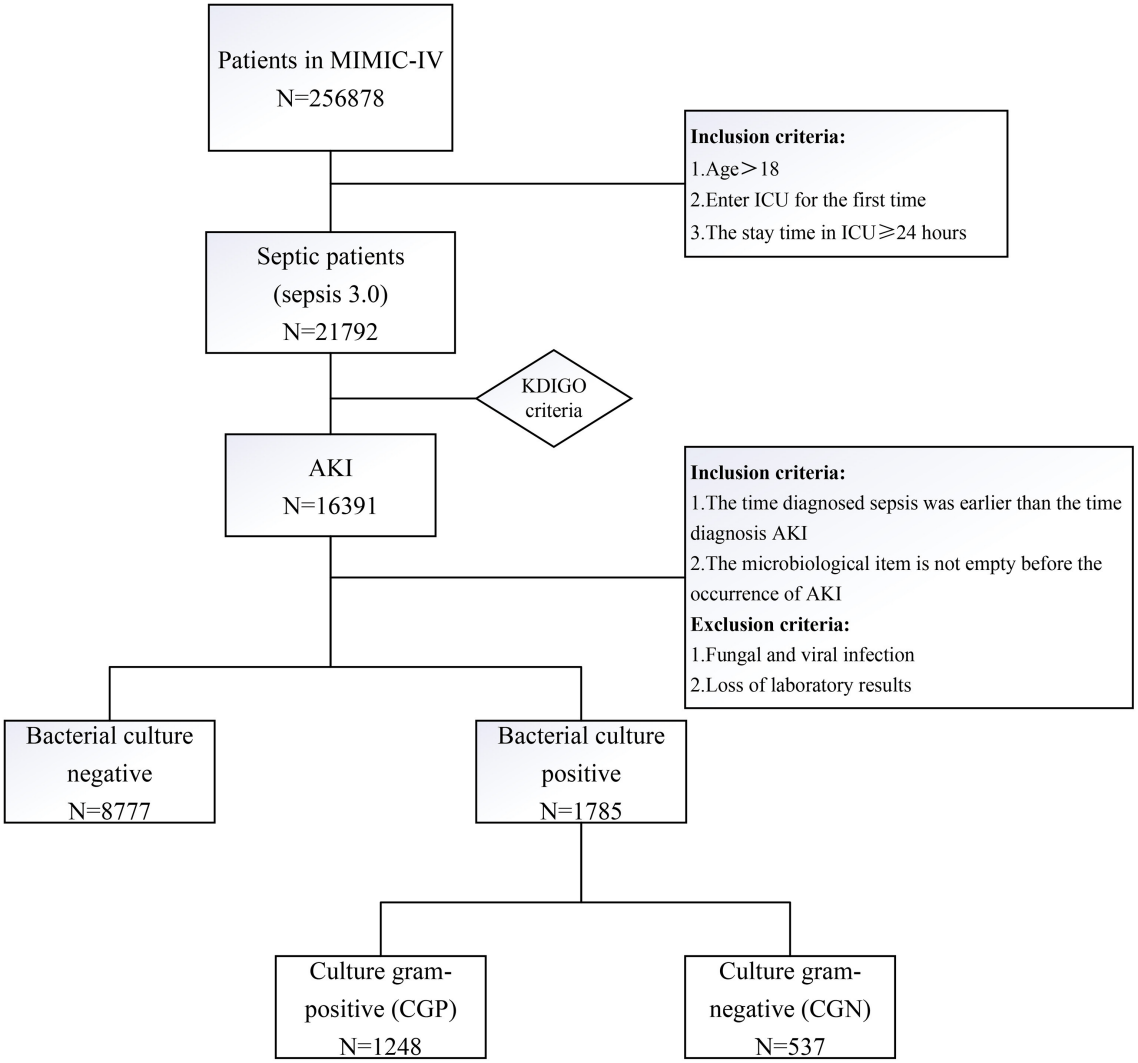
The flowchart of the study population. Data were retrieved from the MIMIC database IV.

There were 256,878 patient records in the MIMIC-IV database, from which we extracted data of 21,792 patients with sepsis. Among them, there were 16,391 patients with AKI-associated sepsis. After excluding the missing data records, 8,777 patients had negative bacterial culture before the occurrence of AKI, and 1,785 patients had positive bacterial culture. The patients with bacterial culture positive were more serious, such as a higher rate of vasopressor use, higher SOFA, and acute physiology (APS) III score. There were more patients in AKI stage III, and had a higher level of creatinine, urea nitrogen, and anion gap. There were more epithelial cells in the urine of patients with positive bacterial culture compared with that in patients with negative bacterial culture. At last, the outcomes of the patients with positive bacterial cultures were worse, such as longer stay in hospital and ICU, and higher ICU and hospital mortality (Supplementary Table 1). Then, 1,785 patients with bacterial culture positive remained and were divided into CGP and CPN groups (Figure 1). The clinical characteristics of each group of patients are shown in Table 1. The CGN group tended to be older (70 vs. 66 years, *p* < 0.001) and had a higher proportion of female patients (49.3 vs. 38.8%, *p* < 0.001). The APSIII score was higher in CGN group (63 vs. 58, *p* = 0.001), however, SOFA scores of the two groups were not significantly different (*p* = 0.079). The CGP group seemed to have higher BMI (28.4 vs. 27, *p* < 0.001), longer time from positive microbial culture to diagnosis of AKI (3.16 vs. 2.94 days, *p* = 0.013). Additionally, the CGN group had longer average ICU stay relative to the CGP group (5.94 vs. 4.77 days, *p* < 0.001). However, there was no significant difference in the CRRT use, ICU mortality, and hospital mortality rates between the two groups (Table 1).

**Table 1:** The baseline characteristics and clinical outcomes of patients with sepsis-associated acute kidney injury (S-AKI) among groups.

**Characteristics**	**Bacterial culture positive**			**Blood culture positive**		
	**CGN**	**CGP**	***P***	**CGNb**	**CGPb**	***P***
	***N*** **= 537**	***N*** **= 1248**		***N*** **= 93**	***N*** **= 264**	
Age (years)	70.0 [57.0; 79.0]	66.0 [55.0; 77.0]	<0.001	69.0 [55.0; 80.0]	62.0 [49.8; 74.2]	0.004
Gender			<0.001			0.292
Female, *n* (%)	265 (49.3%)	484 (38.8%)		46 (49.5%)	112 (42.4%)	
Male, *n* (%)	272 (50.7%)	764 (61.2%)		47 (50.5%)	152 (57.6%)	
BMI (IQR)	27.0 [23.1; 32.5]	28.4 [24.4; 34.3]	<0.001	27.3 [23.9; 34.1]	28.1 [24.3; 33.5]	0.901
Smoker, *n* (%)	62 (11.5%)	125 (10.0%)	0.377	7 (7.53%)	32 (12.1%)	0.304
Alcohol, *n* (%)	41 (7.64%)	120 (9.62%)	0.211	5 (5.38%)	28 (10.6%)	0.197
Vasopressor, *n* (%)	335 (62.4%)	757 (60.7%)	0.526	68 (73.1%)	149 (56.4%)	0.007
SOFA score (IQR)	8.00 [5.00; 11.0]	7.00 [5.00; 10.2]	0.079	10.0 [7.00; 13.0]	9.00 [5.75; 12.0]	0.005
APSIII score (IQR)	63.0 [47.0; 82.0]	58.0 [39.0; 80.0]	0.001	72.0 [56.0; 94.0]	65.5 [50.0; 90.0]	0.114
AKI stage, *n* (%)			0.518			0.514
1	117 (21.8%)	248 (19.9%)		14 (15.1%)	54 (20.5%)	
2	248 (46.2%)	610 (48.9%)		40 (43.0%)	104 (39.4%)	
3	172 (32.0%)	390 (31.2%)		39 (41.9%)	106 (40.2%)	
CKD stage I, *n* (%)	0 (0%)	0 (0%)	.	0 (0%)	0 (0%)	.
CKD stage II, *n* (%)	6 (1.12%)	6 (0.48%)	0.202	2 (2.15%)	2 (0.76%)	0.279
CKD stage III, *n* (%)	28 (5.21%)	57 (4.57%)	0.64	7 (7.53%)	10 (3.79%)	0.16
CKD stage IV, *n* (%)	6 (1.12%)	29 (2.32%)	0.134	1 (1.08%)	4 (1.52%)	1
CKD stage V, *n* (%)	1 (0.19%)	3 (0.24%)	1	1 (1.08%)	0 (0.00%)	0.261
Chronic pulmonary disease, *n* (%)	188 (35.0%)	385 (30.8%)	0.095	24 (25.8%)	69 (26.1%)	1
ARDS, *n* (%)	8 (1.49%)	10 (0.80%)	0.282	1 (1.08%)	4 (1.52%)	1
Hypertension, *n* (%)	275 (51.2%)	615 (49.3%)	0.486	50 (53.8%)	114 (43.2%)	0.101
Heart failure, *n* (%)	65 (12.1%)	176 (14.1%)	0.29	14 (15.1%)	47 (17.8%)	0.656
Diabetes without cc, *n* (%)	139 (25.9%)	339 (27.2%)	0.616	28 (30.1%)	68 (25.8%)	0.498
Diabetes with cc, *n* (%)	44 (8.19%)	127 (10.2%)	0.223	11 (11.8%)	26 (9.85%)	0.733
Creatinine (IQR)	1.2 [0.8; 1.8]	1.2 [0.9; 1.9]	0.409	1.6 [1.0; 2.5]	1.3 [0.9; 2.4]	0.228
Urea nitrogen (IQR)	25.0 [17.0; 42.0]	25.0 [16.0; 42.0]	0.955	29.0 [18.0; 44.0]	32.5 [18.0; 52.0]	0.472
Lactate (IQR)	2.5 [1.6; 4.0]	2.4 [1.7; 3.7]	0.878	3.7 [2.2; 5.5]	2.5 [1.6; 4.4]	0.001
Glucose (IQR)	151.0 [124.0; 207.0]	145.5 [118.0; 202.0]	0.066	163.0 [125.0; 221.0]	158.5 [127.0; 221.2]	0.926
Anion gap (IQR)	17.0 [14.0; 19.0]	16.0 [13.0; 19.0]	0.011	18.0 [16.0; 21.0]	17.0 [14.0; 20.0]	0.017
Epithelial cells (IQR)	76 (14.2%)	145 (11.6%)	0.158	21 (22.6%)	40 (15.2%)	0.14
Total input before AKI diagnosis, (IQR)	3309.8 [1613.5; 7100.5]	3236.9 [1750.3; 5674.3]	0.235	3216.5 [1721.8; 5879.8]	2922.7 [1498.6; 6875.4]	0.868
Total output before AKI diagnosis (IQR)	1640.0 [590.0; 4420.0]	1650.0 [648.0; 3701.8]	0.609	1000.0 [470.0; 1970.0]	1417.5 [273.8; 4168.8]	0.15
Fluid balance before AKI diagnosis (IQR)	1353.2 [125.0; 3093.6]	1235.6 [163.5; 2953.3]	0.64	1943.8 [352.3; 4063.7]	1393.5 [248.2; 3527.6]	0.233
CRRT, *n* (%)	42 (7.82%)	78 (6.25%)	0.266	15 (16.1%)	27 (10.2%)	0.183
Time from positive microbial culture to diagnosis of AKI, *n* (%)	2.94 [1.81; 3.90]	3.16 [2.02; 4.06]	0.013	2.23 [1.64; 3.08]	3.00 [1.87; 4.23]	0.001
Los hospital (IQR)	12.5 [7.66; 20.7]	12.0 [6.97; 20.9]	0.426	13.8 [7.78; 24.9]	14.9 [7.71; 25.9]	0.904
Los ICU (IQR)	5.94 [3.18; 11.0]	4.77 [2.42; 9.13]	<0.001	5.19 [2.75; 11.7]	5.73 [2.95; 9.89]	0.785
Death in ICU, *n* (%)	75 (14.0%)	193 (15.5%)	0.459	13 (14.0%)	60 (22.7%)	0.099
Death in hospital, *n* (%)	107 (19.9%)	264 (21.2%)	0.601	23 (24.7%)	85 (32.2%)	0.224

There were no significant differences between the two groups in kidney function indicators (such as, creatinine, and urea nitrogen), routine blood test results (white blood cell count, platelet count, hemoglobin, etc.), electrolyte indicators (chloride, sodium, etc.), or urine test results (epithelial cells, granular casts, etc.) on the first day after entering the ICU. In the CGN group, the anion gap was higher than that in the CGP group (17 vs. 16, *p* = 0.011).

In our study, the main sites of infection were the urethra, respiratory tract, and blood.

We compared the clinical characteristics and outcomes of blood culture, urine culture, and sputum culture positive patients. The results showed that the patients with blood culture positive had higher SOFA and APSIII score, the ratio of AKI III stage, and the level of creatinine, urea nitrogen, glucose, lactate, anion gap, epithelial cells were higher compared with patients that urine and sputum culture positive. The use rate of CRRT, hospital mortality in the positive blood culture group was much higher than that in the positive urine culture and sputum culture groups. In the positive blood culture group, the time from the positive microbial culture to the diagnosis of AKI was 2.76 days, which was less than that in the positive urine culture (3.32 days) and sputum culture (3.01 days) group (Supplementary Table 2).

We further divided the patients with different sites of infection into Gram-negative bacteria and Gram-positive bacteria groups. In patients with positive blood cultures, the age was older in CGNb group (69 vs. 62 years, *p* = 0.004). They had a higher rate of vasopressor use (73.1 vs. 56.4%, *p* = 0.007), level of lactate (3.7 vs. 2.5, *p* = 0.001), and SOFA score (10 vs. 9, *p* = 0.005) in the CGNb group. The time from positive microbial culture to diagnosis of AKI was shorter than that in the CGPb group (2.23 vs. 3.0 days, *p* = 0.001) (Table 1). In patients with positive sputum culture, the age of CGNs group was elder than that in the CGPs group. And the other indicators had no significant difference between the two groups (Supplementary Table 3). In patients with positive sputum culture, the percentage of female patients in the CGNu group were more than male patients in the CGPu group (Supplementary Table 4).

**Figure 2:**
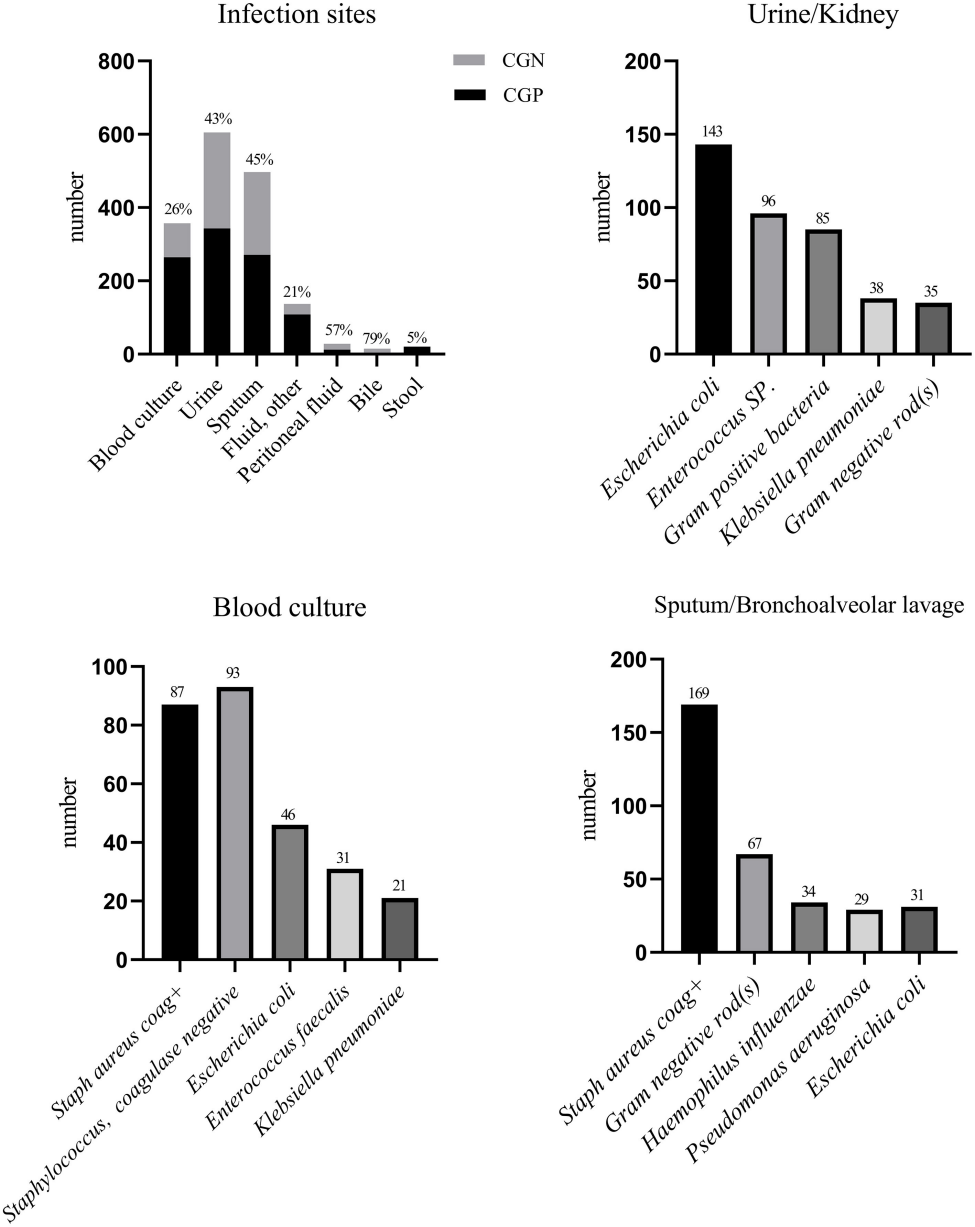
Main sites of infection and the top five bacteria in each site.

Among these infection sites, the number of positive urine cultures was the highest (*N* = 605). Gram-positive bacteria were cultured in 57% of cases, and positive cultures for *Enterococcus sp*. were common. Gram-negative bacteria were cultured in 43% of cases, and *E. coli* had the highest detection rate. The remaining bacteria that were identified included Gram-positive bacteria (no specified name), Gram-negative rod bacteria, and *Klebsiella pneumoniae*. The second most common infection site was the respiratory tract (*N* = 497), and Gram-positive bacteria were cultured in 55% of cases. Coagulase positive (coag+) *S. Aureus* had a high detection rate. Gram-negative bacteria were cultured in 45% of cases. Respiratory cultures mainly included Gram-negative rods (no specified name), *Haemophilus influenzae*-*Beta-Lactamase negative, Pseudomomas Aeruginosa*, and *E. coli* (Figure 2). The third most common site of infection was the blood, and the number of sepsis cases with positive blood cultures was 357. Among these, cultured Gram-positive bacteria accounted for 74% of the total bacteria. The top five bacteria identified in blood cultures were *Staph Aureus Coag*+, *Staphylococcus, Coagulase Negative, E. Coli, Enterococcus Faecalis*, and *Klebsiella Pneumoniae*. Additional sites of infections included peritoneal fluid, other fluids, and bile (Figure 2).

We selected the top five bacteria in blood culture to observe the incidence of septic AKI. The probability of AKI occurring in the five types of bacteria from high to low is as follows: *E. Faecalis* (73.81%), *Staph Aureus Coag*+ (43.5%), *Escherichia Coli* (41.44%), *K. Pneumoniae* (41.18%) and *Staphylococcus, Coagulase Negative* (17.68%) (Supplementary Figure 2).

**Figure 3:**
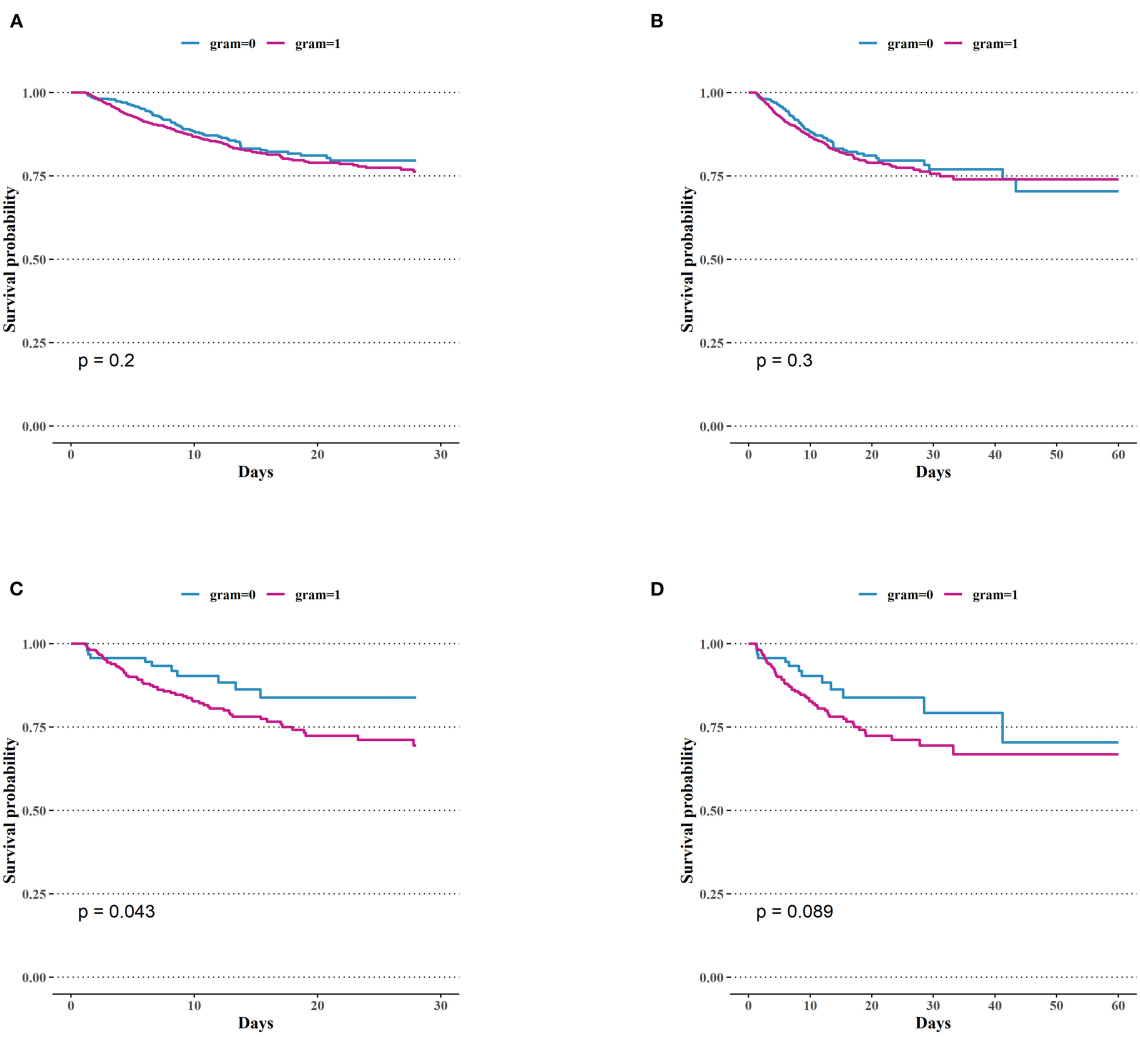
Comparison of Kaplan–Meier survival curves between groups. **(A)** 28-days mortality of intensive care unit (ICU) in patients with bacteria culture Gram-positive and Gram-negative. **(B)** 60-days mortality of ICU in patients with bacteria culture Gram-positive and Gram-negative. **(C)** 28-days mortality in ICU of Gram-negative bacteria group and positive bacteria group in patients with positive blood culture. **(D)** 60-days mortality in ICU of Gram-negative bacteria group and positive bacteria group in patients with positive blood culture.

We analyzed the correlation between variables and ICU mortality in the CGN and CGP groups using the multivariate Cox hazard analysis. In the CGN group, lactate (*HR* = 1.17; 95% *CI* 1.00–1.36, *p* = 0.047), APSIII score (*HR* = 1.02; 95% *CI* 1.01–1.04, *p* = 0.006), los ICU (*HR* = 1.33; 95% *CI* 1.13–1.58, *p* < 0.001), and los hospital (*HR* = 1.02; 95% *CI* 1.01–1.03, *p* = 0.002) were risk factors for ICU mortality. In the CGP group, age (*HR* = 1.01; 95% *CI* 1.00–1.02, *p* = 0.046), APSIII score (*HR* = 1.02; 95% *CI* 1.01–1.02, *p* < 0.001), AKI stage (*HR* = 1.53; 95% *CI* 1.15–2.04, *p* < 0.001), los ICU (*HR* = 1.13; 95% *CI* 1.06–1.20, *p* < 0.001) were risk factors for ICU mortality. In the CGPb group, CRRT (*HR* = 3.48; 95% *CI* 1.35–8.98, *P* = 0.01) was the risk factors for ICU mortality (Table 2). There was no significant difference in the short-term mortality (28 and 60 days) between the CGN and CGP groups (Figures 3A,B). In the blood culture groups, there was significant difference in 28-days survival probability (Figure 3C). There was no significant difference in the 60-day mortality between the two groups (Figure 3D).

**Table 2:** Multivariate Cox regression analysis of the correlation between variables and intensive care unit (ICU) mortality among groups.

**Characteristics**	**Bacterial culture positive**				**Blood culture positive**			
	**CGN** ***N*** **= 537**		**CGP** ***N*** **= 1248**		**CGNb** ***N*** **= 93**		**CGPb** ***N*** **= 264**	
	**HR and CI**	***P***	**HR and CI**	***P***	**HR and CI**	***P***	**HR and CI**	***P***
Age	1.02 (1.00, 1.05)	0.098	1.01 (1.00, 1.02)	0.046	1.03 (0.93, 1.15)	0.556	1.00 (0.99, 1.02)	0.748
Gender = male	1.35 (0.72, 2.54)	0.349	1.13 (0.81, 1.57)	0.482	2.49 (0.28, 22.08)	0.411	1.21 (0.66, 2.21)	0.541
SOFA score	0.92 (0.79, 1.07)	0.252	1.03 (0.95, 1.11)	0.451	1.15 (0.56, 2.38)	0.698	1.09 (0.95, 1.24)	0.221
Lactate	1.17 (1.00, 1.36)	0.047	1.06 (0.99, 1.13)	0.117	0.81 (0.41, 1.60)	0.54	1.03 (0.91, 1.15)	0.661
APS III score	1.02 (1.01, 1.04)	0.006	1.02 (1.01, 1.02)	<0.001	0.99 (0.91, 1.08)	0.852	1.00 (0.99, 1.02)	0.64
AKI stage	1.08 (0.66, 1.76)	0.772	1.53 (1.15, 2.04)	0.004	11.28(0.33,389.45)	0.18	1.64 (0.94, 2.88)	0.083
CRRT	1.43 (0.60, 3.39)	0.423	1.58 (0.97, 2.57)	0.066	4.45 (0.12, 171.78)	0.424	3.48 (1.35, 8.98)	0.01
Los hospital	0.18 (0.13, 0.25)	<0.001	0.53 (0.48, 0.58)	<0.001	0.35 (0.19, 0.63)	<0.001	0.67 (0.59, 0.75)	<0.001
Los ICU	1.33 (1.13, 1.58)	<0.001	1.13 (1.06, 1.20)	<0.001	1.42 (0.94, 2.15)	0.096	1.03 (0.95, 1.13)	0.47

## Discussion

In our study, patients with bacteria culture positive before AKI occurrence had more serious illness compared with patients with bacteria culture negative. In patients with culture positive, results showed that the patients in the CGP group was greater than that in CGN group. But patients in CGN group seemed to be more serious, they were older, had higher APSIII score and long ICU stay time. They also had shorter time to develop into AKI compared with that in the CGP group. However, there was no significant difference in CRRT use, ICU mortality, or hospital mortality between the groups.

Patients with positive blood cultures are more seriously ill compared with other infection sites, also had shorter time to develop into AKI in the CGNb group. Currently, there was many studies focus on the correlation between microbiological analysis and clinical outcomes in patients with sepsis, but a few of study focus on the correlation between bacteria culture types before the onset of AKI and S-AKI outcomes.

It was considered to have SA-AKI or septic AKI that the patients meet consensus criteria for both sepsis and AKI (15). Sepsis and AKI are mutually causal, studies showed that sepsis was associated with up to 50% of AKI, and up to 60% of patients with sepsis have AKI (15, 16). In our study, we retrieved the time of sepsis (SOFA ≥ 2) and AKI (AKI stage was not zero) from the database, and limited qualification that the diagnosis time of sepsis was earlier than the occurrence of AKI.

In 2021, a single-center septic shock study by Kim et al., 1,012 (58.9%) of 1,718 patients with septic shock had positive blood, urine, sputum, or other cultures (17). The number of Gram-negative bacteria present in these cultures was large, which was not completely consistent with our results. In our study, only the patients with S-AKI and the diagnosis time of sepsis earlier than that of AKI were included. There were 1,785 (17%) of 10,562 patients with bacteria positive cultures, and the number of Gram-negative bacteria was 537 (30%). The different results may be because different subjects were included, and more infection sites were analyzed. In our study, there were 87% patients with bacteria negative cultures and had a lighter severity illness, so we did not focus on these patients.

Our study found that in the CGN group, APS III scores, lactate, and los ICU were correlated with the ICU mortality. In the CGP group, age, APS III score, AKI stage, and los ICU were all risk factors. In the patients with blood culture positive, CRRT was the risk factors that correlated with the ICU mortality in CGPb group. Differences between the two groups of patients may be resultant of different pathophysiological mechanisms of sepsis and subsequent AKI.

This study found that there were more patients with positive urine cultures than positive blood or sputum cultures. Gram-negative bacteria were cultured from 43% of urine specimen. Jiang et al. demonstrated that Gram-negative bacteria are the main cause of urethral-associated sepsis. Compared with severe cases, patients with mild sepsis have a higher rate of infection with *E. coli* (18). Ramakrishnan et al. showed that in more than 80% of acute pyelonephritis cases, the causative pathogen is *E. coli*; other common causes include aerobic Gram-negative bacteria, *Staphylococcus saprophyticus*, and *Enterococcus* (19). In our analysis, we found that, except for *E. coli*, bacterial species that were identified from positive urine cultures were *Enterococcus*, Gram-positive and Gram-negative bacteria not specifically named, *K. pneumoniae*, etc., which are roughly the same species. Although there is no clear conclusion on whether *E. coli* infection of the urethra is related to sepsis AKI, Wang et al. showed that alpha-hemolysin of uropathogenic *E. coli* induces GM-CSF-mediated AKI (20). Shi et al. used urine-derived sepsis to create a septic AKI model (21) and demonstrated that *E. coli* infection of the urethra is related to AKI. However, the specific mechanism underlying this relationship is unknown and requires further research.

At present, blood cultures are a significant clinical diagnostic tool. Studies regarding blood culture results and patient outcomes have mainly focused on sepsis and septic shock with no emphasis on AKI. International guidelines for the management of sepsis and septic shock recommend analyzing blood cultures before initiating anti-infective treatment (22), so that targeted treatment can be implemented. A FABLED Cohort Study in 2021 showed that the time of blood culture positive is unrelated to the mortality of patients with severe sepsis (23). A meta-analysis performed by Li et al. showed that culture positivity or negativity was not associated with mortality in sepsis or septic shock patients, and that culture positive and culture-negative sepsis patients have similar lengths of ICU stay, mechanical ventilation requirements, and renal replacement requirements. In our study, we analyzed the probability of AKI occurring in the top 5 bacterial species in blood culture, and found that the rate of AKI happened in the patients with *E. Faecalis* positive cultures was higher than others. Currently, there was few research on the correlation of *E. Faecalis* infection in blood culture and the occurrence of AKI. The study found that *E. Faecalis* has tropism for the kidneys in the urinary tracts of mice (24). In 2015, Sarai Little Ibrahim et al. found that in the Children with *E. Faecalis* bacteremia, receiving low-dose gentamicin had ~2 times the risk of developing AKI than that were not receiving this agent (25). This suggested that the use of antibacterial drugs may play an important role in the occurrence of AKI in children with *E. Faecalis* bacteremia, and related mechanisms need to be further explored in the future.

The occurrence of *S. aureus* coag^+^ in sputum cultures may be related to the use of ventilators or severe lung diseases. AKI is common in cases of community-acquired pneumonia and is associated with an increased risk of death (26). In this clinical study, the 28- and 90-day survival data were not available, and the impact on long-term prognosis has not yet been analyzed. Therefore, our conclusions are somewhat limited. Additionally, bronchoalveolar lavage fluid was included in the sputum cultures analyzed in this study. Therefore, it was impossible to distinguish upper and lower respiratory tract infections and corresponding bacterial species from these specimens.

In our study, we did not judge whether the bacteria are pathogenic based on the disease process, accompanying diseases, microbial culture susceptibility results, and antibiotic application. This is a limitation of this study.

## Conclusions

The Gram types of bacteria cultured prior to S-AKI occurrence was not related to AKI stage, CRRT application, and short-term mortality. Compared with Gram-positive bacterial infections, Gram-negative bacterial infections take a shorter time to develop into AKI, and had a higher disease severity score.

## Data Availability Statement

The original contributions presented in the study are included in the article/Supplementary Material, further inquiries can be directed to the corresponding authors.

## Ethics Statement

MIMIC-IV database access was approved by the Massachusetts Institute of Technology (Cambridge, MA, USA) and Beth Israel Deaconess Medical Center (Boston, MA, USA). Consent to participate was obtained during the original data collection.

## Author Contributions

AL and MD contributed to the conception and design. TL contributed to the collection and assembly of data and data analysis and interpretation. All authors contributed to manuscript writing and final approval of manuscript.

## Conflict of Interest

The authors declare that the research was conducted in the absence of any commercial or financial relationships that could be construed as a potential conflict of interest.

## Publisher's Note

All claims expressed in this article are solely those of the authors and do not necessarily represent those of their affiliated organizations, or those of the publisher, the editors and the reviewers. Any product that may be evaluated in this article, or claim that may be made by its manufacturer, is not guaranteed or endorsed by the publisher.

## Abbreviations

AKI: acute kidney injury
CGN: culture Gram-negative
CGP: culture Gram-positive
CGNb: culture Gram-negative bacteria in patients with positive blood cultures
CGPb: culture Gram-positive bacteria in patients with positive blood cultures
CRRT: continuous renal replacement therapy
ICU: intensive care unit
BMI: body mass index
ARDS: acute respiratory distress syndrome
Los ICU: length of ICU stay time
Los hospital: length of hospital stay time
Diabetes with cc: diabetes with complication
Diabetes without cc: diabetes without complication
SOFA: Sequential Organ Failure Assessment.
