# Supplementary material for: The Correlation Between the Types of Initial Bacterial Infection and Clinical Prognosis in Patients With Septic AKI
Source: Front Med (Lausanne). 2022 Jan 27;8:800532. doi: 10.3389/fmed.2021.800532 (PMC8828919; doi:10.3389/fmed.2021.800532)
Supplement: Supplementary file 1 [file Table_1.DOCX]

| Characteristics | Total | Culture Positive | Culture Negative | *P* |
| --- | --- | --- | --- | --- |
|  | N=10562 | N=1785 | N=8777 |  |
| Age (years) | 68.0 [58.0;79.0] | 67.0 [56.0;78.0] | 68.0 [58.0;79.0] | <0.001 |
| Gender |  |  |  | 0.05 |
| Female | 4210 (39.9%) | 749 (42.0%) | 3461 (39.4%) |  |
| Male | 6352 (60.1%) | 1036 (58.0%) | 5316 (60.6%) |  |
| BMI | 28.2 [24.6;33.1] | 28.0 [24.1;33.9] | 28.3 [24.7;32.9] | 0.32 |
| Smoker | 996 (9.43%) | 187 (10.5%) | 809 (9.22%) | 0.106 |
| Alcohol | 844 (7.99%) | 161 (9.02%) | 683 (7.78%) | 0.087 |
| Vasopressor | 5873 (55.6%) | 1092 (61.2%) | 4781 (54.5%) | <0.001 |
| SOFA score | 6.00 [4.00;9.00] | 7.00 [5.00;11.0] | 6.00 [4.00;9.00] | <0.001 |
| APSIII score | 52.0 [37.0;73.0] | 60.0 [41.0;81.0] | 51.0 [36.0;72.0] | <0.001 |
| AKI stage |  |  |  | <0.001 |
| 1 | 2482 (23.5%) | 365 (20.4%) | 2117 (24.1%) |  |
| 2 | 5273 (49.9%) | 858 (48.1%) | 4415 (50.3%) |  |
| 3 | 2807 (26.6%) | 562 (31.5%) | 2245 (25.6%) |  |
| CKD stage I | 4 (0.04%) | 0 (0.00%) | 4 (0.05%) | 1 |
| CKD stage II | 57 (0.54%) | 12 (0.67%) | 45 (0.51%) | 0.508 |
| CKD stage III | 488 (4.62%) | 85 (4.76%) | 403 (4.59%) | 0.802 |
| CKD stage IV | 156 (1.48%) | 35 (1.96%) | 121 (1.38%) | 0.08 |
| CKD stage V | 33 (0.31%) | 4 (0.22%) | 29 (0.33%) | 0.616 |
| Chronic pulmonary  disease | 2839 (26.9%) | 573 (32.1%) | 2266 (25.8%) | <0.001 |
| ARDS | 67 (0.63%) | 18 (1.01%) | 49 (0.56%) | 0.043 |
| Hypertension | 5510 (52.2%) | 890 (49.9%) | 4620 (52.6%) | 0.034 |
| Heart failure | 1299 (12.3%) | 241 (13.5%) | 1058 (12.1%) | 0.097 |
| Diabetes without cc | 2625 (24.9%) | 478 (26.8%) | 2147 (24.5%) | 0.042 |
| Diabetes with cc | 876 (8.29%) | 171 (9.58%) | 705 (8.03%) | 0.035 |
| Creatinine (IQR) | 1.1 [0.8;1.7] | 1.2 [0.9;1.9] | 1.1 [0.8;1.7] | 0.001 |
| Urea nitrogen (IQR) | 22.0 [16.0;36.0] | 25.0 [16.0;42.0] | 22.0 [16.0;35.0] | <0.001 |
| Lactate (IQR) | 2.5 [1.7;3.8] | 2.4 [1.7;3.8] | 2.5 [1.7;3.8] | 0.064 |
| Glucose (IQR) | 144.0 [118.0;192.0] | 148.0 [120.0;203.0] | 143.0 [117.0;190.8] | 0.002 |
| Anion gap (IQR) | 15.0 [13.0;19.0] | 16.0 [13.0;19.0] | 15.0 [13.0;19.0] | <0.001 |
| Epithelial cells (IQR) | 1047 (9.9%) | 221 (12.4%) | 826 (9.4%) | <0.001 |
| Total input before AKI diagnosis, (IQR) | 2939.9 [1549.3;4849.8] | 3260.5  [1710.9;6029.2] | 2881.8 [1520.0;4667.7] | <0.001 |
| Total output before AKI diagnosis (IQR) | 1702.0 [639.2;3398.0] | 1650.0  [607.0;3961.0] | 1720.0 [640.0;3330.0] | 0.14 |
| Fluid balance before AKI diagnosis (IQR) | 988.2 [-69.8;2298.1] | 1268.5  [149.3;2966.7] | 943.8 [-103.1;2183.0] | <0.001 |
| CRRT | 569 (5.39%) | 120 (6.72%) | 449 (5.12%) | 0.007 |
| Los hospital | 9.01 [5.69;15.7] | 12.1 [7.05;20.9] | 8.61 [5.42;14.6] | <0.001 |
| Los ICU | 3.60 [2.06;7.08] | 5.08 [2.58;9.75] | 3.34 [1.99;6.56] | <0.001 |
| Death in ICU | 1237 (11.7%) | 268 (15.0%) | 969 (11.0%) | <0.001 |
| Death in hospital | 1681 (15.9%) | 371 (20.8%) | 1310 (14.9%) | <0.001 |

Supplementary table 1. Baseline characteristics of S-AKI patients between groups of bacterial culture positive and negative.
