# Supplementary material for: The Correlation Between the Types of Initial Bacterial Infection and Clinical Prognosis in Patients With Septic AKI
Source: Front Med (Lausanne). 2022 Jan 27;8:800532. doi: 10.3389/fmed.2021.800532 (PMC8828919; doi:10.3389/fmed.2021.800532)
Supplement: Supplementary file 2 [file Table_2.DOCX]

| **Characteristics** | **Total** | **Blood culture** | **sputum culture** | **Urine culture** | ***P*** | **blood *vs* sputum** | **blood *vs* urine** | **sputum *vs* urine** |
| --- | --- | --- | --- | --- | --- | --- | --- | --- |
|  | **N=1459** | **N=357** | **N=497** | **N=605** |  | ***P*** | ***P*** | ***P*** |
| Age (years) | 67.0 [54.0;78.0] | 64.0 [52.0;77.0] | 64.0 [50.0;76.0] | 71.0 [59.0;79.0] | <0.001 | 0.52 | <0.001 | <0.001 |
| Gender |  |  |  |  | <0.001 | 0.069 | 0.018 | <0.001 |
| Female | 666 (45.6%) | 158 (44.3%) | 188 (37.8%) | 320 (52.9%) |  |  |  |  |
| Male | 793 (54.4%) | 199 (55.7%) | 309 (62.2%) | 285 (47.1%) |  |  |  |  |
| BMI | 27.3 [23.6;33.3] | 28.1 [24.2;33.8] | 26.6 [23.0;31.8] | 27.9 [23.7;34.3] | 0.003 | 0.006 | 0.641 | 0.006 |
| Smoker | 163 (11.2%) | 39 (10.9%) | 71 (14.3%) | 53 (8.76%) | 0.015 | 0.269 | 0.323 | 0.016 |
| Alcohol | 144 (9.87%) | 33 (9.24%) | 60 (12.1%) | 51 (8.43%) | 0.118 | 0.347 | 0.754 | 0.173 |
| Vasopressor | 873 (59.8%) | 217 (60.8%) | 270 (54.3%) | 386 (63.8%) | 0.006 | 0.105 | 0.387 | 0.005 |
| SOFA score | 8.00 [5.00;11.0] | 9.00 [6.00;12.0] | 8.00 [6.00;11.0] | 7.00 [5.00;11.0] | <0.001 | 0.023 | <0.001 | 0.009 |
| APSIII score | 63.0 [46.0;85.0] | 68.0 [52.0;92.0] | 65.0 [48.0;85.0] | 60.0 [40.0;81.0] | <0.001 | 0.012 | <0.001 | 0.007 |
| AKI stage |  |  |  |  | 0.002 | 0.003 | 0.01 | 0.269 |
| 1 | 296 (20.3%) | 68 (19.0%) | 113 (22.7%) | 115 (19.0%) |  |  |  |  |
| 2 | 688 (47.2%) | 144 (40.3%) | 243 (48.9%) | 301 (49.8%) |  |  |  |  |
| 3 | 475 (32.6%) | 145 (40.6%) | 141 (28.4%) | 189 (31.2%) |  |  |  |  |
| Chronic pulmonary disease | 487 (33.4%) | 93 (26.1%) | 191 (38.4%) | 203 (33.6%) | 0.001 | 0.001 | 0.027 | 0.106 |
| ARDS | 19 (1.30%) | 5 (1.40%) | 9 (1.81%) | 5 (0.83%) | 0.325 | 0.847 | 0.769 | 0.712 |
| Hypertension | 700 (48.0%) | 164 (45.9%) | 230 (46.3%) | 306 (50.6%) | 0.245 | 0.977 | 0.278 | 0.278 |
| Heart failure | 199 (13.6%) | 61 (17.1%) | 51 (10.3%) | 87 (14.4%) | 0.013 | 0.015 | 0.302 | 0.074 |
| Diabetes without cc | 380 (26.0%) | 96 (26.9%) | 109 (21.9%) | 175 (28.9%) | 0.029 | 0.167 | 0.546 | 0.03 |
| Diabetes with cc | 135 (9.25%) | 37 (10.4%) | 29 (5.84%) | 69 (11.4%) | 0.005 | 0.031 | 0.695 | 0.005 |
| Creatinine (IQR) | 1.2 [0.8;2.0] | 1.4 [1.0;2.4] | 1.1 [0.8;1.7] | 1.2 [0.9;2.0] | <0.001 | <0.001 | 0.015 | 0.001 |
| Urea nitrogen (IQR) | 27.0 [17.0;46.0] | 32.0 [18.0;50.0] | 23.0 [15.0;42.0] | 27.0 [17.0;46.0] | <0.001 | <0.001 | 0.085 | 0.005 |
| Lactate (IQR) | 2.5 [1.6;4.0] | 2.6 [1.6;4.9] | 2.3 [1.5;3.7] | 2.5 [1.8;3.9] | 0.002 | 0.003 | 0.15 | 0.022 |
| Glucose (IQR) | 153.0 [122.0;209.0] | 159.0 [126.0;221.0] | 156.0 [126.0;207.0] | 146.0 [118.0;206.0] | 0.004 | 0.264 | 0.005 | 0.031 |
| Anion gap (IQR) | 17.0 [14.0;20.0] | 17.0 [15.0;20.0] | 16.0 [14.0;19.0] | 16.0 [14.0;19.0] | <0.001 | 0.007 | <0.001 | 0.189 |
| Epithelial cells (IQR) | 233 (16.0%) | 61 (17.1%) | 73 (14.7%) | 99 (16.4%) | 0.603 | 0.746 | 0.84 | 0.746 |
| Total input before AKI diagnosis, (IQR) | 3423.8 [1691.9;7818.1] | 3069.7 [1543.5;6646.9] | 5019.2 [2144.7;11905.9] | 3069.6 [1549.9;5584.2] | <0.001 | <0.001 | 0.399 | <0.001 |
| Total output before AKI diagnosis (IQR) | 1740.0 [580.5;5236.5] | 1210.0 [300.0;3410.0] | 3060.0 [900.0;8885.0] | 1615.0 [545.0;3590.0] | <0.001 | <0.001 | 0.027 | <0.001 |
| Fluid balance before AKI diagnosis (IQR) | 1355.1 [168.0;3348.5] | 1540.5 [285.0;3570.2] | 1619.7 [158.7;4070.7] | 1068.4 [120.6;2778.8] | 0.002 | 0.869 | 0.007 | 0.005 |
| CRRT | 116 (7.95%) | 42 (11.8%) | 26 (5.23%) | 48 (7.93%) | 0.002 | 0.002 | 0.095 | 0.096 |
| Time micro to AKI | 3.11 [1.95;4.19] | 2.76 [1.81;3.90] | 3.01 [1.93;4.16] | 3.32 [2.17;4.29] | 0.001 | 0.152 | <0.001 | 0.026 |
| Los hospital | 13.3 [7.67;21.9] | 14.8 [7.77;25.8] | 14.8 [8.58;23.7] | 11.6 [7.11;19.6] | <0.001 | 0.825 | 0.001 | <0.001 |
| Los ICU | 6.04 [3.18;11.5] | 5.70 [2.90;10.1] | 8.50 [4.74;14.6] | 4.80 [2.42;8.63] | <0.001 | <0.001 | 0.006 | <0.001 |
| Death in ICU | 265 (18.2%) | 73 (20.4%) | 98 (19.7%) | 94 (15.5%) | 0.088 | 0.86 | 0.123 | 0.123 |
| Death in hospital | 359 (24.6%) | 108 (30.3%) | 120 (24.1%) | 131 (21.7%) | 0.011 | 0.084 | 0.011 | 0.363 |

Supplementary table 2. Baseline characteristics of S-AKI patients among blood culture, urine culture and sputum culture groups. The p value of pairwise comparison between multiple groups was corrected by BH method.
