# Supplementary material for: The Correlation Between the Types of Initial Bacterial Infection and Clinical Prognosis in Patients With Septic AKI
Source: Front Med (Lausanne). 2022 Jan 27;8:800532. doi: 10.3389/fmed.2021.800532 (PMC8828919; doi:10.3389/fmed.2021.800532)
Supplement: Supplementary file 3 [file Table_3.DOCX]

| Characteristics | Total | CGN(s) | CGP(s) | *P* |
| --- | --- | --- | --- | --- |
|  | N=497 | N=226 | N=271 |  |
| Age (year) | 64.0 [50.0;76.0] | 69.0 [57.0;77.0] | 60.0 [43.0;73.5] | <0.001 |
| gender |  |  |  | 0.354 |
| Female, n (%) | 188 (37.8%) | 80 (35.4%) | 108 (39.9%) |  |
| Male, n (%) | 309 (62.2%) | 146 (64.6%) | 163 (60.1%) |  |
| BMI (IQR) | 26.6 [23.0;31.8] | 26.4 [22.9;31.5] | 26.7 [23.4;32.2] | 0.395 |
| Smoker, n (%) | 71 (14.3%) | 37 (16.4%) | 34 (12.5%) | 0.278 |
| Alcohol, n (%) | 60 (12.1%) | 21 (9.29%) | 39 (14.4%) | 0.11 |
| vasopressor, n (%) | 270 (54.3%) | 126 (55.8%) | 144 (53.1%) | 0.622 |
| SOFA score (IQR) | 8.00 [6.00;11.0] | 8.00 [5.00;11.0] | 8.00 [6.00;11.0] | 0.11 |
| APSIII score (IQR) | 65.0 [48.0;85.0] | 65.0 [51.0;83.8] | 63.0 [46.0;86.0] | 0.866 |
| AKI stage, n (%) |  |  |  | 0.052 |
| 1 | 113 (22.7%) | 57 (25.2%) | 56 (20.7%) |  |
| 2 | 243 (48.9%) | 97 (42.9%) | 146 (53.9%) |  |
| 3 | 141 (28.4%) | 72 (31.9%) | 69 (25.5%) |  |
| CKD stage I, n (%) | 0 (0%) | 0 (0%) | 0 (0%) | . |
| CKD stage II, n (%) | 3 (0.60%) | 1 (0.44%) | 2 (0.74%) | 1 |
| CKD stage III, n (%) | 19 (3.82%) | 10 (4.42%) | 9 (3.32%) | 0.686 |
| CKD stage IV, n (%) | 6 (1.21%) | 3 (1.33%) | 3 (1.11%) | 1 |
| CKD stage V, n (%) | 2 (0.40%) | 1 (0.44%) | 1 (0.37%) | 1 |
| Chronic pulmonary disease, n (%) | 191 (38.4%) | 96 (42.5%) | 95 (35.1%) | 0.109 |
| ARDS, n (%) | 9 (1.81%) | 5 (2.21%) | 4 (1.48%) | 0.738 |
| hypertension, n (%) | 230 (46.3%) | 107 (47.3%) | 123 (45.4%) | 0.73 |
| Heart failure, n (%) | 51 (10.3%) | 25 (11.1%) | 26 (9.59%) | 0.698 |
| Diabetes without cc, n (%) | 109 (21.9%) | 56 (24.8%) | 53 (19.6%) | 0.196 |
| Diabetes with cc, n (%) | 29 (5.84%) | 14 (6.19%) | 15 (5.54%) | 0.904 |
| Creatinine (IQR) | 1.1 [0.8;1.7] | 1.1 [0.8;1.7] | 1.0 [0.8;1.6] | 0.69 |
| Urea nitrogen (IQR) | 23.0 [15.0;42.0] | 23.0 [17.0;44.0] | 23.0 [14.5;41.0] | 0.312 |
| Lactate (IQR) | 2.3 [1.5;3.7] | 2.3 [1.5;3.4] | 2.4 [1.5;4.0] | 0.31 |
| Glucose (IQR) | 156.0 [126.0;207.0] | 162.0 [128.2;213.0] | 154.0 [123.0;203.5] | 0.237 |
| Anion gap (IQR) | 16.0 [14.0;19.0] | 16.0 [14.0;19.0] | 16.0 [14.0;20.0] | 0.605 |
| Epithelial cells (IQR) | 73 (14.7%) | 31 (13.7%) | 42 (15.5%) | 0.666 |
| Total input before AKI diagnosis, (IQR) | 5019.2 [2144.7;11905.9] | 4884.8 [1939.0;10934.6] | 5521.8 [2310.1;12953.8] | 0.158 |
| Total output before AKI diagnosis (IQR) | 3060.0 [900.0;8885.0] | 2771.0  [771.0;8532.0] | 3510.0 [985.0;10157.5] | 0.184 |
| Fluid balance before AKI diagnosis (IQR) | 1619.7 [158.7;4070.7] | 1402.7  [113.5;3599.0] | 1820.0 [261.1;4248.4] | 0.155 |
| CRRT, n (%) | 26 (5.23%) | 15 (6.64%) | 11 (4.06%) | 0.279 |
| Time micro to AKI (IQR) | 3.01 [1.93;4.16] | 2.84 [1.76;3.96] | 3.18 [2.10;4.24] | 0.023 |
| Los hospital (IQR) | 14.8 [8.58;23.7] | 13.9 [8.60;22.8] | 15.2 [8.52;24.6] | 0.57 |
| Los ICU (IQR) | 8.50 [4.74;14.6] | 8.29 [4.91;14.7] | 8.63 [4.51;14.4] | 0.905 |
| Death in ICU, n (%) | 98 (19.7%) | 44 (19.5%) | 54 (19.9%) | 0.989 |
| Death in hospital, n (%) | 120 (24.1%) | 55 (24.3%) | 65 (24.0%) | 1 |

Supplementary Table 3. Among patients with positive sputum culture, the baseline characteristics comparison between CGNs and CGPs groups.
