# Supplementary material for: The Correlation Between the Types of Initial Bacterial Infection and Clinical Prognosis in Patients With Septic AKI
Source: Front Med (Lausanne). 2022 Jan 27;8:800532. doi: 10.3389/fmed.2021.800532 (PMC8828919; doi:10.3389/fmed.2021.800532)
Supplement: Supplementary file 4 [file Table_4.DOCX]

| Characteristics | Total | CGN(u) | CGP(u) | *P* |
| --- | --- | --- | --- | --- |
|  | N=605 | N=262 | N=343 |  |
| Age (years) | 71.0 [59.0;79.0] | 72.0 [59.0;80.0] | 70.0 [60.0;79.0] | 0.203 |
| Gender |  |  |  | <0.001 |
| Female, n (%) | 320 (52.9%) | 161 (61.5%) | 159 (46.4%) |  |
| Male, n (%) | 285 (47.1%) | 101 (38.5%) | 184 (53.6%) |  |
| BMI (IQR) | 27.9 [23.7;34.3] | 27.2 [23.5;33.9] | 28.4 [24.1;34.4] | 0.25 |
| Smoker, n (%) | 53 (8.76%) | 20 (7.63%) | 33 (9.62%) | 0.477 |
| Alcohol, n (%) | 51 (8.43%) | 18 (6.87%) | 33 (9.62%) | 0.29 |
| Vasopressor, n (%) | 386 (63.8%) | 174 (66.4%) | 212 (61.8%) | 0.279 |
| SOFA score (IQR) | 7.00 [5.00;11.0] | 7.00 [5.00;10.0] | 8.00 [5.00;11.0] | 0.75 |
| APSIII score (IQR) | 60.0 [40.0;81.0] | 60.0 [43.0;80.0] | 60.0 [39.0;82.5] | 0.588 |
| AKI stage, n (%) |  |  |  | 0.273 |
| 1 | 115 (19.0%) | 57 (21.8%) | 58 (16.9%) |  |
| 2 | 301 (49.8%) | 129 (49.2%) | 172 (50.1%) |  |
| 3 | 189 (31.2%) | 76 (29.0%) | 113 (32.9%) |  |
| Chronic pulmonary disease, n (%) | 203 (33.6%) | 89 (34.0%) | 114 (33.2%) | 0.918 |
| ARDS, n (%) | 5 (0.83%) | 2 (0.76%) | 3 (0.87%) | 1 |
| Hypertension, n (%) | 306 (50.6%) | 139 (53.1%) | 167 (48.7%) | 0.326 |
| Heart failure, n (%) | 87 (14.4%) | 37 (14.1%) | 50 (14.6%) | 0.967 |
| Diabetes without cc, n (%) | 175 (28.9%) | 79 (30.2%) | 96 (28.0%) | 0.623 |
| Diabetes with cc, n (%) | 69 (11.4%) | 25 (9.54%) | 44 (12.8%) | 0.258 |
| Creatinine (IQR) | 1.2 [0.9;2.0] | 1.2 [0.9;1.9] | 1.3 [0.9;2.2] | 0.13 |
| Urea nitrogen (IQR) | 27.0 [17.0;46.0] | 26.0 [17.0;43.8] | 28.0 [17.0;48.5] | 0.154 |
| Lactate (IQR) | 2.5 [1.8;3.9] | 2.4 [1.6;4.0] | 2.6 [1.8;3.8] | 0.127 |
| Glucose (IQR) | 146.0 [118.0;206.0] | 148.0 [121.2;212.0] | 143.0 [116.0;202.5] | 0.326 |
| Anion gap (IQR) | 16.0 [14.0;19.0] | 16.0 [14.0;19.0] | 16.0 [14.0;20.0] | 0.32 |
| Epithelial cells (IQR) | 99 (16.4%) | 41 (15.6%) | 58 (16.9%) | 0.761 |
| Total input before AKI diagnosis, (IQR) | 3069.6 [1549.9;5584.2] | 3032.4 [1420.1;5361.8] | 3078.4 [1690.7;5918.7] | 0.386 |
| Total output before AKI diagnosis (IQR) | 1615.0 [545.0;3590.0] | 1550.0 [578.8;3607.5] | 1675.0 [518.0;3547.5] | 0.725 |
| Fluid balance before AKI diagnosis (IQR) | 1068.4 [120.6;2778.8] | 981.7 [30.5;2716.3] | 1216.1 [238.2;2874.1] | 0.375 |
| CRRT, n (%) | 48 (7.93%) | 19 (7.25%) | 29 (8.45%) | 0.696 |
| Time micro to AKI | 3.32 [2.17;4.29] | 3.31 [1.95;4.14] | 3.32 [2.31;4.47] | 0.084 |
| Los hospital | 11.6 [7.11;19.6] | 11.3 [7.22;16.8] | 11.7 [7.01;20.9] | 0.387 |
| Los ICU | 4.80 [2.42;8.63] | 4.67 [2.76;8.72] | 4.86 [2.40;8.59] | 0.782 |
| Death in ICU, n (%) | 94 (15.5%) | 34 (13.0%) | 60 (17.5%) | 0.16 |
| Death in hospital, n (%) | 131 (21.7%) | 50 (19.1%) | 81 (23.6%) | 0.215 |

Supplementary table 4. Among patients with positive urine culture, the baseline characteristics comparison between CGNu and CGPu groups.
