# Supplementary figures and images for: The Correlation Between the Types of Initial Bacterial Infection and Clinical Prognosis in Patients With Septic AKI
Source: Front Med (Lausanne). 2022 Jan 27;8:800532. doi: 10.3389/fmed.2021.800532 (PMC8828919; doi:10.3389/fmed.2021.800532)

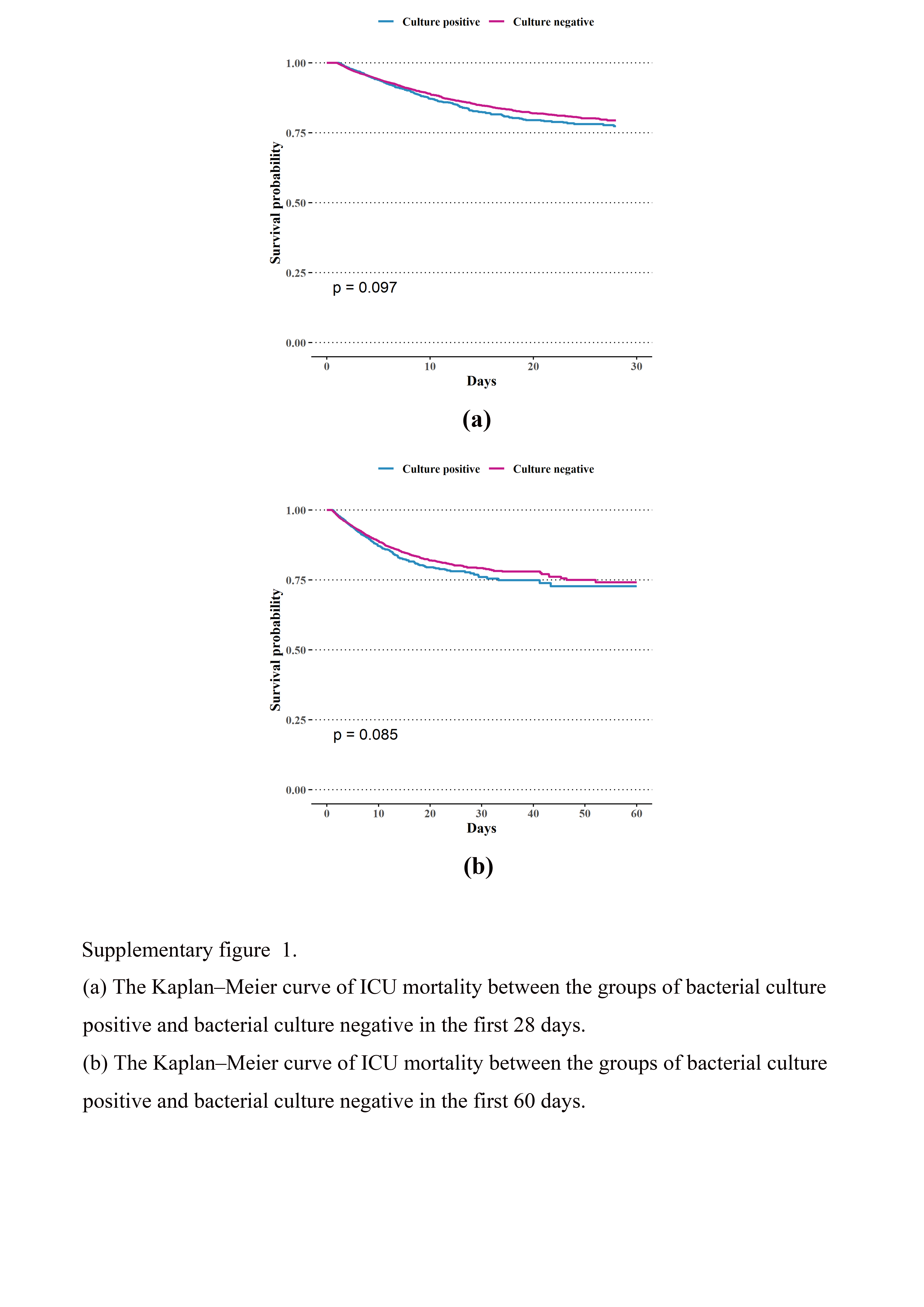

Supplement: Supplementary file 5 [file Image_1.TIF]

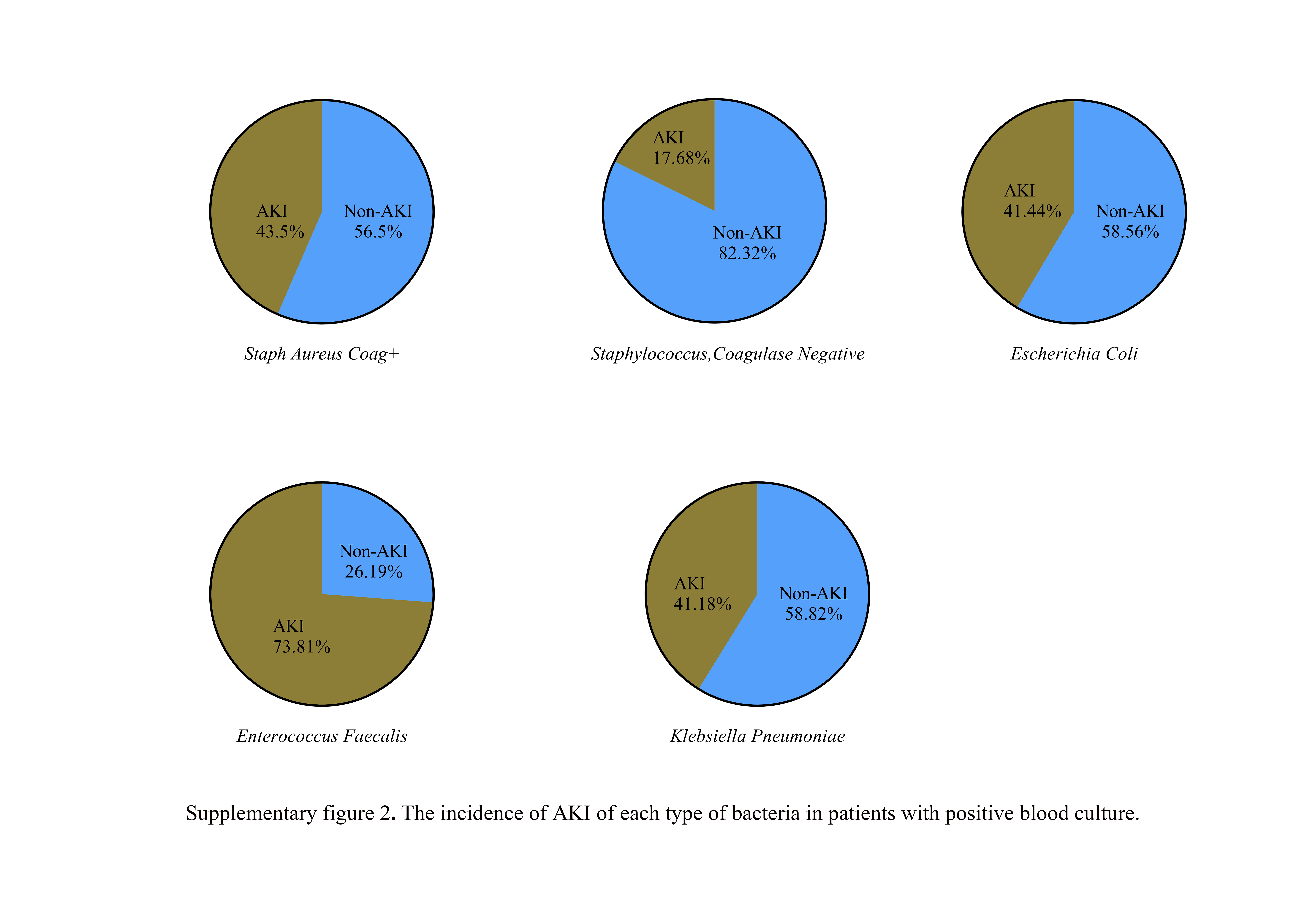

Supplement: Supplementary file 6 [file Image_2.TIF]
